# Raynaud and digital ulcers in patients with juvenile systemic sclerosis: ambulatory iloprost protocol. A single center experience

**DOI:** 10.1186/1546-0096-12-S1-P308

**Published:** 2014-09-17

**Authors:** Margarida Guedes, Carla Zilhão, Isabel Almeida, Ivone Silva

**Affiliations:** 1 Pediatric Department, Porto Hospital Center, Porto, Portugal; 2 Clinical Immunology Unit, Porto Hospital Center, Porto, Portugal; 3 Vascular Surgey Department, Porto Hospital Center, Porto, Portugal

## Introduction

Raynaud Phenomenon (RP) and digital ulcers (DU) are the main clinical manifestation of vasculopathy secondary to Juvenile Systemic Sclerosis (jSSc) . Accepted treatment for RP is calcium channel blockers. Iloprost is indicated if active ulcers are present or if RP is refractory to treatment.

## Objectives

The aim of this study was to access the safety and efficacy of ambulatory treatment with intravenous prostanoides in children with RP and DU secondary to jSSc**.**

## Methods

Three patients with jSSc and active digital ulcers were treated with intravenous iloprost . Our protocol establishes two treatments in the cold months: one in the beginning and the second 3 months later, of a continuous five day intravenous infusion(iv) of iloprost in a dose of 0.4- 0,6 mg/kg/min perfusion through an elastomeric pump.

## Results

Since 2007 we treated three patients with jSSc and active digital ulcers: two girls, 12 and 13 years, and a boy of 18 months old at time of first treatment. A total of 19 treatments of iv. iloprost were done, 15 of these in ambulatory basis with the elastomeric pump

Outcomes were good with a reduced number and severity of RP attacks in all patients, and reduced pain associated with RP and DU. No disabling or severe side effects were observed. Two patients, the girls, had no more active digital ulcers after the second treatment with a follow-up of 2 and 3 years respectively. One patient, male, has recurrent digital ulcers, but number has decreased from 4 to 1 not interfering with daily activities.

## Conclusion

Iloprost iv. perfusion with the elastomeric pump at ambulatory is a safe and effective treatment for patients with refractory RP to calcium channel blockers and in patients with digital ulcers. The authors suggest two treatments in the winter season with reduction of number and severity of R and ulcer healing.

## Disclosure of interest

None declared.

